# 176 Reducing travel distances in Sport – good for people and the planet

**DOI:** 10.1093/eurpub/ckae114.108

**Published:** 2024-09-26

**Authors:** Karim Abu-Omar, Dogukan Özer, Antonina Tcymbal, Tobias Volk

**Affiliations:** Friedrich-Alexander Universität Erlangen-Nürnberg, Erlangen, Germany; Friedrich-Alexander Universität Erlangen-Nürnberg, Erlangen, Germany; Friedrich-Alexander Universität Erlangen-Nürnberg, Erlangen, Germany; Friedrich-Alexander Universität Erlangen-Nürnberg, Erlangen, Germany

## Abstract

**Purpose:**

Globally, about 30% of CO2 emissions are caused by travel. In order to comply with calls of the United Nations and the European Commission, the sport sector needs to reduce its emissions caused by travel to tournaments and league games in professional and amateur sport. To this date, no clear mechanisms exists on how to do this. The presentations shows how an algorithm based solution from optimisation science can help sport federations reducing travel distances in any type of league or tournament play.

**Methods:**

The sport federations of American Football (16 adolescent teams), Biathlon (IBU World Cup Series) and Gymnastics (64 1st and 2nd division Faustball teams) provided data on team locations, league play and tournament formats. Locations of teams and tournaments were mapped with Google Maps, and travel distances (straight-line, by car) were analysed. The algorithm Gurobi from optimisation science was used to optimise league play and tournaments to minimise total travel distances.

**Results:**

For American Football, 16 adolescent teams travel in total 6329 km (396 km per team) for a season of league play in 2024. The IBU World Cup featuring 10 events for the 2023/24 season results in 13466 km of travel for each participant. In both instances, the optimisation algorithm can propose significant reductions in travel distances. For the IBU World Cup, changing the order of tournaments can reduce travel distances by more than 30%. The analysis of Faustball data is ongoing.

**Conclusions:**

Travel distances in amateur and professional sport are a significant source of CO2 emissions. Algorithm based optimisation of league and tournament play to reduce travel distances has good potential to put the sport sector more in line with CO2 reduction targets agreed upon in the Paris Agreement.

**Support/Funding Source:**

No external funds.

